# Artificial neural network-boosted Cardiac Arrest Survival Post-Resuscitation In-hospital (CASPRI) score accurately predicts outcome in cardiac arrest patients treated with targeted temperature management

**DOI:** 10.1038/s41598-022-11201-z

**Published:** 2022-05-04

**Authors:** Szu-Yi Chou, Oluwaseun Adebayo Bamodu, Wei-Ting Chiu, Chien-Tai Hong, Lung Chan, Chen-Chih Chung

**Affiliations:** 1 Graduate Institute of Neural Regenerative Medicine, College of Medical Science and Technology, Taipei Medical University, Taipei, Taiwan, ROC; 2 Ph.D. Program for Neural Regenerative Medicine, College of Medical Science and Technology, Taipei Medical University and National Health Research Institutes, Taipei, Taiwan, ROC; 3 Department of Medical Research & Education, Shuang Ho Hospital, Taipei Medical University, New Taipei City, 235 Taiwan, ROC; 4 Department of Urology, Shuang Ho Hospital, Taipei Medical University, New Taipei City, 235 Taiwan, ROC; 5 Department of Hematology & Oncology, Shuang Ho Hospital, Taipei Medical University, New Taipei City, 235 Taiwan, ROC; 6 Department of Neurology, Shuang Ho Hospital, Taipei Medical University, 291, Zhongzheng Road, Zhonghe District, New Taipei City, 235 Taiwan, ROC; 7 Department of Neurology, School of Medicine, College of Medicine, Taipei Medical University, Taipei City, 110 Taiwan, ROC; 8 Division of Critical Care Medicine, Department of Emergency and Critical Care Medicine, Taipei Medical University-Shuang Ho Hospital, New Taipei City, 235 Taiwan, ROC; 9 Graduate Institute of Biomedical Informatics, College of Medical Science and Technology, Taipei Medical University, Taipei City, 110 Taiwan, ROC

**Keywords:** Network models, Machine learning, Prognosis, Therapeutics, Hypoxic-ischaemic encephalopathy

## Abstract

Existing prognostic models to predict the neurological recovery in patients with cardiac arrest receiving targeted temperature management (TTM) either exhibit moderate accuracy or are too complicated for clinical application. This necessitates the development of a simple and generalizable prediction model to inform clinical decision-making for patients receiving TTM. The present study explores the predictive validity of the Cardiac Arrest Survival Post-resuscitation In-hospital (CASPRI) score in cardiac arrest patients receiving TTM, regardless of cardiac event location, and uses artificial neural network (ANN) algorithms to boost the prediction performance. This retrospective observational study evaluated the prognostic relevance of the CASPRI score and applied ANN to develop outcome prediction models in a cohort of 570 patients with cardiac arrest and treated with TTM between 2014 and 2019 in a nationwide multicenter registry in Taiwan. In univariate logistic regression analysis, the CASPRI score was significantly associated with neurological outcome, with the area under the receiver operating characteristics curve (AUC) of 0.811. The generated ANN model, based on 10 items of the CASPRI score, achieved a training AUC of 0.976 and validation AUC of 0.921, with the accuracy, precision, sensitivity, and specificity of 89.2%, 91.6%, 87.6%, and 91.2%, respectively, for the validation set. CASPRI score has prognostic relevance in patients who received TTM after cardiac arrest. The generated ANN-boosted, CASPRI-based model exhibited good performance for predicting TTM neurological outcome, thus, we propose its clinical application to improve outcome prediction, facilitate decision-making, and formulate individualized therapeutic plans for patients receiving TTM.

## Introduction

Cardiac arrest is implicated in a notable proportion of premature deaths and disabilities worldwide^1–3^. A substantial proportion of patients treated for out-of-hospital cardiac arrest (OHCA) die before emergency department arrival, and a significant proportion of in-hospital cardiac arrest (IHCA) patients-associated deaths occur during the initial resuscitation. Of those who have restoration of spontaneous circulation (ROSC) after initial resuscitation, a large proportion die before discharge, and only a minority of patients with cardiac arrest achieve favorable functional outcome at discharge^1,4^. However, it is noteworthy that several factors, including patients’ age, pre-arrest neurological condition, functional status, rhythm type (shockable *vs* non-shockable) play vital role in the outcome of OHCA^5,6^. In context of these outcome predictors, we note that of the minority of patients who are discharged alive with favorable functional status, the proportion sdischarged with favorable neurological status is relatively high^6^, and this may explain the attribution of majority of OHCA post-resuscitation deaths to brain injury^7^, howbeit without accounting for deaths due to early withdrawal of life-sustaining treatment which in itself frequently truncates the opportunity for brain recovery^8^.

To prevent or reduce the poor outcomes associated with cardiac arrest, targeted temperature management (TTM) has been introduced and touted to reduce mortality and improve the neurological recovery in patients with cardiac arrest^9–12^.

Several prognostic factors associated with the outcome in cardiac arrest patients treated with TTM have been identified, and a number of tools have been proposed for outcome prognostication^13–17^. So far, these prognostic and/or predictive models either exhibit moderately acceptable accuracy, are designed exclusively for OHCA patients, or are too complicated with multiple clinical variables for the clinical applications^13–16^. This necessitates the development of a simple and yet generalizable prediction model to inform clinical decision-making and formulation of therapeutic strategies for patients receiving or indicated for TTM with different clinical status.

The Cardiac Arrest Survival Post-resuscitation In-hospital (CASPRI) score consisting of eleven items, was designed to predict clinical outcome of patients who achieve ROSC after experiencing IHCA^18^, and has been validated in different cohorts with good discrimination power reported^18,19^. However, its predictive performance in a non-selective patient cohort who received TTM regardless of place of cardiac arrest (IHCA and OHCA) has not been validated. In the present study, we hypothesized that the CASPRI score is clinically applicable in predicting the outcome in cardiac arrest patients treated with TTM, regardless of place of event.

Advances in machine learning algorithms coupled with increased computational power continue to enable enhanced diagnostic and prognostic capabilities in various medical fields. Recently published reports suggest the capability of artificial neural networks (ANN), a supervised machine learning algorithm, to accurately predict neurological outcomes, including survival, for patients with OHCA or IHCA^15,33,34^.

The present study explored the clinical validity of the CASPRI score in patients with cardiac arrest, regardless of place of event, who received TTM, and proffered improvement of the predictive accuracy of the CASPRI score by applying ANN-based prediction models.

## Materials and methods

### Participants

This retrospective cohort study used clinical data from medical records obtained from the Taiwan Network of Targeted Temperature Management for Cardiac Arrest (TIMECARD) registry^14^. TIMECARD registry is a nationwide multicenter registry project conducted from January 2014 and September 2019 in 9 medical centers in Taiwan. An on-line case report form was built for every participating hospital to report their patient-level data. All electronic medical data was decoupled from patient identifying information.

The inclusion criteria for the TIMECARD registry were: (1) participants aged 18 years or older, (2) a cardiac event occurring inside or outside the hospital, (3) receipt of cardiopulmonary resuscitation (CPR) with ROSC, (4) Glasgow coma scale (GCS) less than 8 or inability to obey commands after ROSC, and (5) receipt of TTM less than 12 h after ROSC.

The exclusion criteria were as follows: patients with (1) uncontrollable bleeding, (2) impaired consciousness before cardiac arrest or pre-cardiac arrest, indicated by cerebral performance category (CPC) score ≤ 3, regardless of etiology, (3) fatal ventricular arrhythmia (tachycardia or fibrillation), (4) intracranial hemorrhage, or (5) life expectancy less than 6 months.

All eligible patients were treated using the TTM protocol consistent with the consensus of scientific statement from the Taiwan Society of Emergency & Critical Care Medicine^12^. The variables were retrieved from archived patients’ registry data based on the updated Utstein Resuscitation Registry template, and included baseline characteristics, comorbidities, coupled with information on the cardiac arrest event, etiology, post-arrest care, and the outcomes^12,14,20^. Modeled after the CASPRI score development and validation studies, which to the best of our knowledge, employed retrospective determination of pre- and post-arrest CPC score for predicting neurological outcomes for patients with cardiac arrest, the CPC score in our study was retrospectively determined from patients’ information garnered from family members and/or medical records by the research investigator, who is also consultant neurologist in each medical center^14,19–22^.

A favorable neurological outcome was defined as CPC score of 1–2 (conscious and alert with good or moderate cerebral performance) at the time of discharge, while poor outcome was defined as CPC score of 3–5 (severe neurological disability, persistent vegetative state, or death)^12,14,20–22^.

### Ethical approval

The study was approved by the Joint Institutional Review Board of Taipei Medical University (TMU-JIRB Approval No. N201711046). Waiver of informed consent were approved by the TMU-JIRB for this retrospective study involving the secondary analysis of existing anonymized data. All methods were performed in accordance with the relevant guidelines and regulations.

### Statistical analyses

All analyses were performed using JMP^®^ version14.2.0 (SAS Institute Inc., Cary, NC, USA). Variables were summarized using descriptive statistics. Continuous variables are presented as mean ± standard deviation, and categorical variables are expressed as counts and percentages. One-way ANOVA was used to determine the statistical significance of differences between means of ≥ 3 independent variables and Fisher’s exact test to determine non-random associations between 2 categorical variables. A two-tailed *p*-value of < 0.05 was considered statistically significant.

### Application of CASPRI score

**Figure 1:**
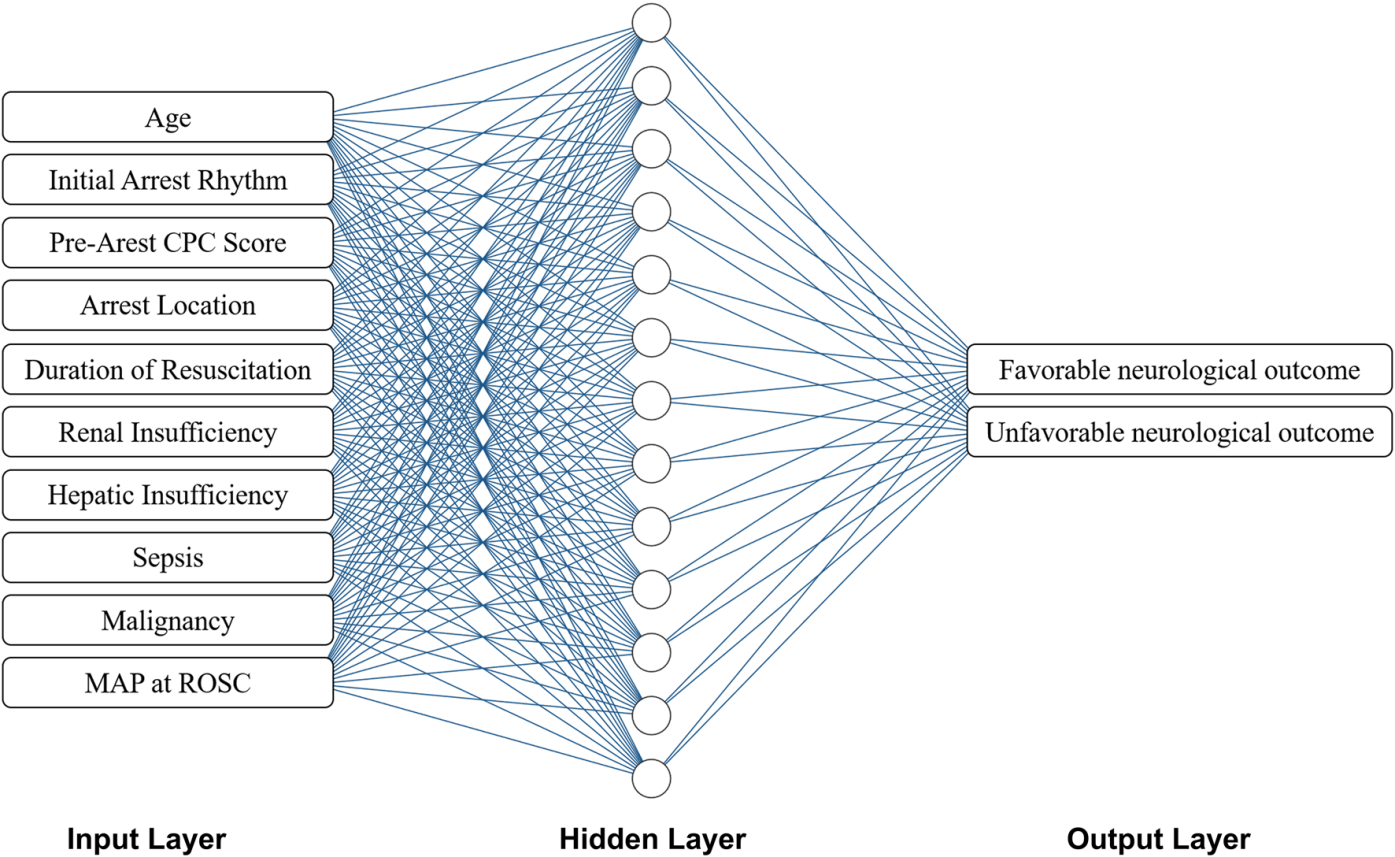
Artificial neural network (ANN) model in the present study. Schema showing the input, hidden, and output layers of the ANN model. The number of neurons in the hidden layer were set empirically and ranged from 1 to 50. The output layer contains two neurons—the favorable and unfavorable neurological outcome at hospital discharge. *ANN* artificial neural network, *CPC* cerebral performance category, *MAP* mean arterial pressure, *ROSC* restoration of spontaneous circulation.

The CASPRI score (Table S1) was calculated for each patient as earlier described by Chan et al. in the original development and internal validation study^18^. In TIMECARD registry, all clinical information of interventions prior to the time of cardiac arrest were excluded^14^, thus, mechanical ventilation, indicated as one variable of the CASPRI score^18^, was not included in the analysis. To calculate the CASPRI score for OHCA patients, arrest location was scored 3 points, being the score for patients from non-monitored unit^18^. Overall, 10 out of the 11 items of the CASPRI score were incorporated into our logistic regression and ANN models (Table 1, Fig. 1).

**Table 1:** Baseline demographic characteristics of patients according to neurological outcomes at hospital discharge.

Variables	Whole cohort (n = 570)	Favorable outcome (n = 117)	Unfavorable outcome (n = 453)	*p*-value	OR (95% CI)
Age (years)^a^	64.6 ± 15.9	58.1 ± 16.6	66.3 ± 15.3	< 0.0001	1.03 (1.02–1.05)^b^
Female, n (%)	194 (34.0)	30 (25.6)	164 (36.2)	0.037	0.61 (0.38–0.96)
**Initial cardiac arrest rhythm, n (%)**^**a**^				< 0.0001	
VF/Pulseless VT	209 (36.7)	79 (67.5)	130 (28.7)		
Pulseless electrical activity	137 (24.0)	30 (26.6)	107 (23.6)		
Asystole	224 (39.3)	8 (6.8)	216 (47.7)		
Pre-arrest CPC score^a^	1.29 ± 0.60	1.04 ± 0.20	1.36 ± 0.65	< 0.0001	6.46 (2.7–15.4)^b^
**Arrest location, n (%)**^**a**^					
OHCA	463 (81.2)	97 (82.9)	366 (80.8)	0.691	1.15 (0.68–1.97)
IHCA	107 (18.8)	20 (17.1)	87 (19.2)	0.250	
Telemetry unit	57 (10.0)	14 (12.0)	43 (9.5)		
Intensive care unit	9 (1.6)	1 (0.9)	8 (1.8)		
Non-monitored unit	41 (7.2)	5 (4.3)	36 (8.0)		
Duration of resuscitation (min)^a^	24.0 ± 17.7	21.5 ± 21.0	24.7 ± 16.7	0.132	1.01 (1.0–1.02)^b^
MAP at ROSC (mmHg)^a^	94.6 ± 31.0	104.3 ± 29.8	92.2 ± 30.8	0.0001	0.99 (0.98–0.99)^b^
**Comorbidities, n (%)**					
Renal insufficiency^a^	144 (25.3)	18 (15.4)	126 (27.8)	0.006	0.47 (0.27–0.81)
Hepatic insufficiency^a^	18 (3.2)	1 (0.9)	17 (3.8)	0.142	0.22 (0.03–1.68)
Sepsis^a^	59 (10.4)	3 (2.6)	56 (12.4)	0.001	0.19 (0.06–0.61)
Malignancy^a^	72 (12.6)	7 (6.0)	65 (14.3)	0.013	0.38 (0.17–0.85)
Diabetes mellitus	236 (41.4)	33 (28.2)	203 (44.8)	0.001	0.48 (0.31–0.75)
Hypertension	322 (56.5)	62 (53.0)	260 (57.4)	0.404	0.84 (0.56–1.26)
Coronary artery disease	152 (26.7)	30 (25.6)	122 (26.9)	0.816	0.94 (0.59–1.49)
Heart failure	109 (19.1)	17 (14.5)	92 (20.3)	0.187	0.66 (0.38–1.17)
Arrhythmia	71 (12.5)	16 (13.7)	55 (12.1)	0.640	1.15 (0.63–2.08)
COPD or asthma	62 (10.9)	6 (5.1)	56 (12.4)	0.029	0.38 (0.16–0.91)
Previous cerebral vascular disease	74 (13.0)	6 (5.1)	68 (15.0)	0.003	0.31 (0.13–0.72)
CASPRI score	17.8 ± 5.6	13.2 ± 3.8	18.9 ± 5.5	< 0.0001	1.28 (1.21–1.36)^b^

*CASPRI* Cardiac Arrest Survival Postresuscitation In-hospital, *CI* confidence interval, *COPD* chronic obstructive pulmonary disease, *CPC* cerebral performance category, *IHCA* in-hospital cardiac arrest, *MAP* mean arterial pressure, *OHCA* out-of-hospital cardiac arrest, *OR* odds ratio, *ROSC* return of spontaneous circulation, *VF* ventricular fibrillation, *VT* ventricular tachycardia.

^a^Variables used to calculate CASPRI score.

^b^Odds ratio of per unit changes.

Univariate logistic regression analysis, with the CASPRI score considered a continuous variable, was performed to determine probable association between the total score and the outcome. We assessed our regression model using area under the receiver operating characteristics curve (AUC), with the accuracy, precision, sensitivity, and specificity of the univariate logistic regression model indicated.

### Development and validation of ANN models

The ANN model was developed using STATISTICA ver. 13.3 (TIBCO Software Inc., Tulsa, Oklahoma, USA). The applied ANN architecture was a multilayer perceptron, containing an input layer, one hidden layer, and an output layer (Fig. 1). Continuous variables included age, pre-arrest CPC score, duration of resuscitation, and mean arterial pressure (MAP) at ROSC. Categorical variables included initial arrest rhythm, arrest location, renal or hepatic insufficiency, sepsis, and malignancy. The arrest locations were indicated as four independent input neurons in the ANN model, namely, OHCA, telemetry unit, intensive care unit, and non-monitored unit. The numbers of neurons in the hidden layer were set empirically, ranging from 1 to 50.

### Oversampling of the minority classes

To reduce the disproportionate ratio of patients with favorable neurological outcomes to those with unfavorable outcomes in the dataset, we applied the Synthetic Minority Over-sampling Technique (SMOTE) to the minority class, namely the subset of favorable neurological outcomes^23^. By analyzing samples in minority class and synthesizing new samples based on them, SMOTE can improve classification performance and help circumvent limitations associated with overly skewed or imbalanced data, thus enhancing the accuracy and generalizability of the prediction model^23^. By the SMOTE, 336 samples of favorable neurological outcomes were synthetically oversampled to re-balance the class distribution (Table S2). After oversampling, 453 samples each of favorable and unfavorable neurological outcomes were randomly partitioned into 80% training and 20% validation sets in the ANN models while maintaining an identical proportion of favorable and unfavorable outcomes.

### Model evaluation

The generalizability of the analysis was assessed using five-fold cross-validation. The model performance was evaluated using five independent validation sets. The mean AUC of the five training and validation sets and the mean accuracy, precision, sensitivity, and specificity of the five validation sets are reported.

## Results

### Cohort demographics and baseline characteristics

A total of 580 patients were registered in the TIMECARD database. Ten patients without documented CPC score at discharge were excluded from the analysis. Overall, 570 patients (194 female and 376 male; mean age 64.6 ± 15.9 years) who received TTM treatment were eligible and enrolled into this study. Among them, there were 463 (81.2%) patients with OHCA and 107 (18.8%) with IHCA. At hospital discharge, 117 (20.5%) patients had favorable neurological outcomes, and 453 (79.5%) patients had unfavorable neurological outcomes. The mortality rate was 59.1% (n = 337). Compared to those with unfavorable outcomes, patients with favorable neurological outcomes at hospital discharge were younger, had lower CPC score 24 h before cardiac arrest, and higher MAP at ROSC. More so, patients with favorable neurological outcomes were more prone to ventricular fibrillation/pulseless ventricular tachycardia, less likely in asystole during initial cardiac arrest, or exhibit renal insufficiency, sepsis, malignant disease, or other systemic/chronic diseases (Table 1).

### The association between CASPRI score and the outcomes

The mean CASPRI score was 17.8 ± 5.6 points for the whole cohort, and the score was significantly higher in patients with unfavorable neurological status at hospital discharge (18.9 ± 5.5 vs 13.2 ± 3.8) (Table 1). Unadjusted binary regression analysis showed that every point increase in the CASPRI score was associated with 1.28-fold (95% CI 1.21–1.36; p < 0.0001) or 1.14-fold (95% CI 1.10–1.18; p < 0.0001) increase in the likelihood of an unfavorable outcome or mortality outcome, respectively, for cardiac arrest patients who received TTM.

**Figure 2:**
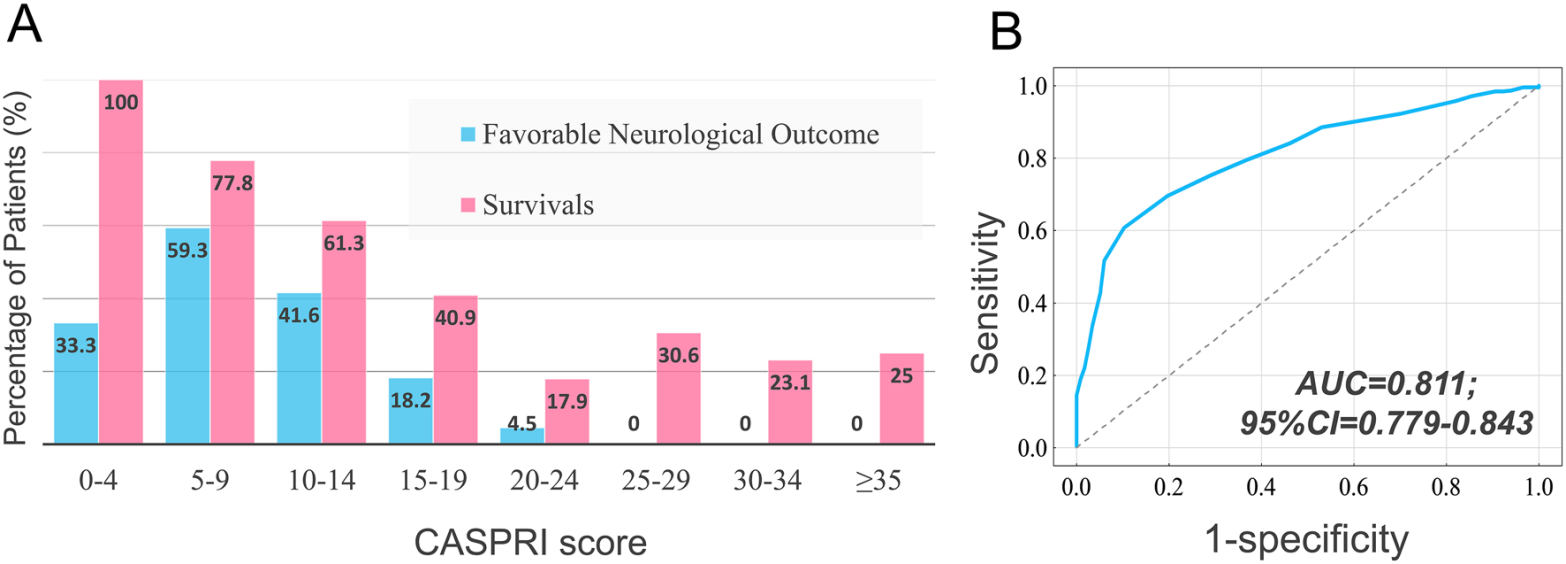
Predictive performance of the CASPRI score. (**A**) Graphical visualization of the corresponding percentage of cardiac arrest patients who received TTM in current cohort who survived to hospital discharge and who had favorable neurological outcomes for every 5-points increases of CASPRI score. (**B**) ROC curve with indicated AUC of the CASPRI score univariable logistic regression model to predict favorable neurological outcomes in cardiac arrest patients who received TTM. *AUC* area under the curve, *ROC* receiver operating characteristic, *CASPRI* Cardiac Arrest Survival Post-resuscitation In-hospital, *TTM* targeted temperature management.

For the patients with CASPRI score < 10, there was a 56.7% probability of favorable neurological outcome and 80.0% probability of survival at discharge, while patients with CASPRI score ≥ 25 had no chance of a favorable neurological outcome (Fig. 2A). As shown in Fig. 2B, the AUC of CASPRI score to predict favorable outcome was 0.811; 95% CI 0.779–0.843, with the accuracy, precision, sensitivity, and specificity of 79.8%, 52.5%, 17.9%, and 95.8%, respectively. The results indicated that the original CASPRI score exhibits good specificity but relatively low sensitivity in the prediction of neurological outcomes for the patients with cardiac arrest who received TTM.

### Boosting the predictive performance of CASPRI score by using ANN

**Figure 3:**
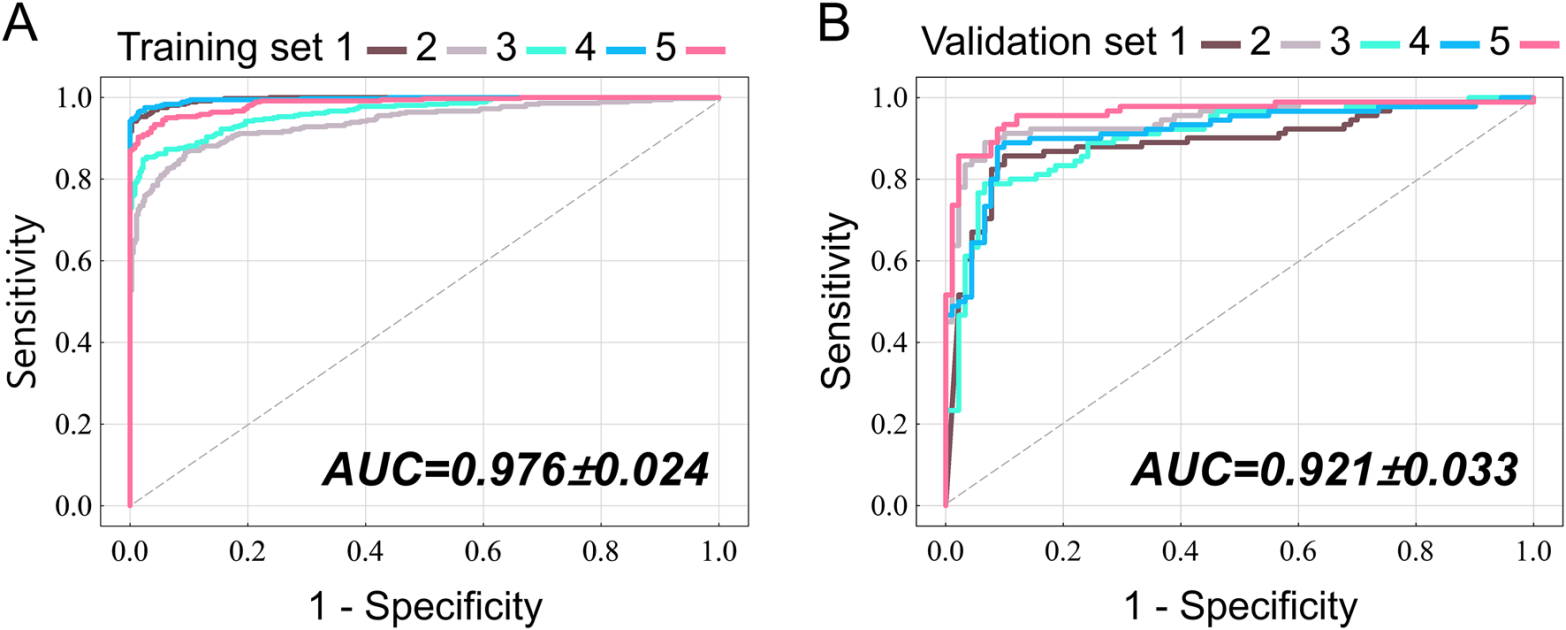
Predictive performance of ANN models. ROC curves with AUCs of the (**A**) training and (**B**) validation sets of ANN model to predict favorable neurological outcomes in cardiac arrest patients who received TTM using baseline parameters of CASPRI score. AUC values are presented as mean ± SD of the five training and validation sets during five-fold cross-validation. *ANN* artificial neural network, *AUC* area under the curve, *ROC* receiver operating characteristic, *CASPRI* Cardiac Arrest Survival Post-resuscitation In-hospital, *TTM* targeted temperature management, *SD* standard deviation.

As alluded earlier, ten baseline characteristics from the items of CASPRI score were used as the input attributes to develop the ANN model for predicting neurological outcomes. After adequate training, the ANN-boosted CASPRI models containing 8, 27, 45, 46, and 47 hidden neurons achieved the best prediction performance for the fivefold cross-validation sets, with a mean training accuracy of 93.5 ± 3.8% and validation accuracy of 89.2 ± 2.5%. The precision of the validation set was 91.6 ± 1.3%, sensitivity was 87.6 ± 4.2%, and specificity was 91.2 ± 1.1%. The AUC was 0.976 ± 0.024 for the training set (Fig. 3A) and 0.921 ± 0.033 for the validation set (Fig. 3B).

A comparative analysis of the predictive performance of the original CASPRI score and ANN-boosted models were performed. As shown in Table 2, the results indicate that the ANN models achieved relatively higher accuracy, precision, sensitivity, and AUC values in predicting favorable neurological outcomes, with improved accuracy when predicting the clinical outcomes of patients with cardiac arrest who received TTM.

**Table 2:** Comparison of the performance of CASPRI score and ANN-boosted CASPRI model for predicting functional outcomes of patients received TTM.

Model	Accuracy	Precision	Sensitivity	Specificity	AUC
CASPRI score	0.798	0.525	0.179	**0.958**	0.811
ANN-boosted CASPRI model	**0.892**	**0.916**	**0.876**	0.912	**0.921**

Univariate logistic regression analysis was performed using the CASPRI score as a continuous variable to calculated the AUC. The higher value among the two models is shown in bold.

*ANN* artificial neural network, *AUC* area under the receiver operating characteristic curve, *CASPRI* Cardiac Arrest Survival Postresuscitation In-hospital.

### Relative significance of predictors

**Figure 4:**
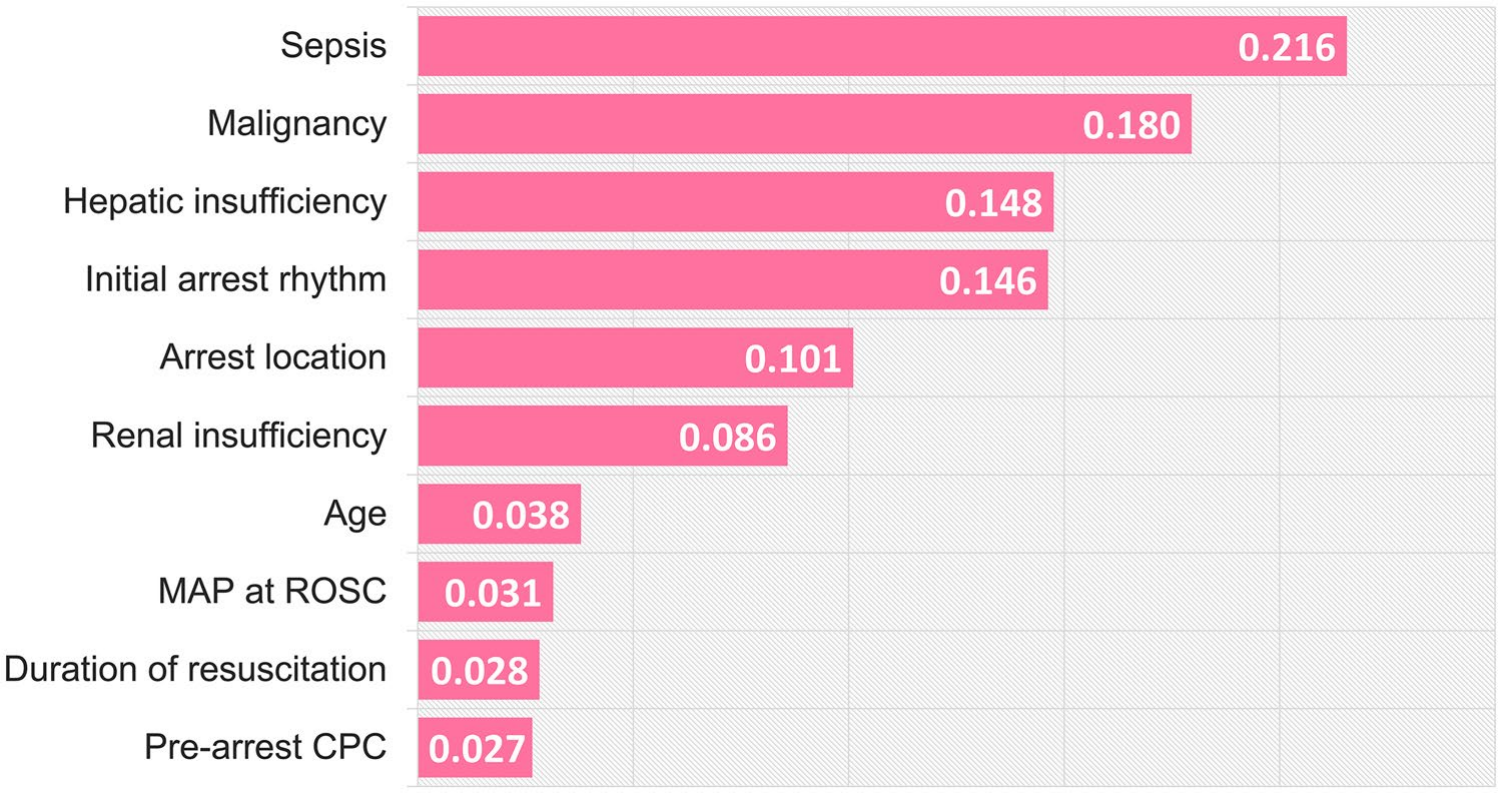
Significance of variables in the ANN model. Graphical representation of the relative significance of the individual parameters in the ANN model. The numbers in each color-coded bar indicate the calculated indices of the total effect of the predicting factors, with a higher value representing a greater significance attributed to the model. *ANN* artificial neural network, *CPC* cerebral performance category, *MAP* mean arterial pressure, *ROSC* restoration of spontaneous circulation.

A sensitivity analysis was performed to assess the predictive value of each parameter in the ANN model, and evaluated the relative contribution of that parameter alone and in combination with other factors in the model. The relative significance of each factor was indicated by its mean importance value through five repetitions during five-fold cross-validation. Among all parameters of the CASPRI score, sepsis, malignancy, hepatic insufficiency, initial cardiac arrest rhythm, and arrest location were the strongest predictors of neurological outcomes (Fig. 4). These results provide some insight into the significance of the parameters that contribute to neurological prognosis of cardiac arrest patients who were treated with TTM.

## Discussion

The present study validates the CASPRI score and the application of ANN-based models to predict or boost prediction of clinical outcomes in patients who received TTM. The CASPRI score exhibited prognostic relevance with an AUC of 0.811 to predict favorable neurological outcomes in patients who received TTM after cardiac arrest. Interestingly, the established ANN-boosted CASPRI score model achieved better predictive performance with an AUC of 0.921 for predicting the neurological outcomes in the validation set. This is of relevance for precision medicine, because the AUC measures the degree of discriminability between groups, thus, the relatively higher AUC values indicate that the ANN-boosted models bode well for patient stratification, and can distinguish the groups of interest, namely favorable *versus* unfavorable neurological outcomes in cardiac arrest patients who received TTM.

The CASPRI score was initially developed using successfully resuscitated IHCA patients—a population in which prognostication is particularly helpful in making decisions regarding the intensity of life support and associated management strategy^18^. Chan et al. in their study, reported CASPRI score with an AUC of 0.802 for predicting favorable neurological outcomes^18^. This score has been validated using patients of East Asian descent, wherein the AUC for CASPRI score was 0.77–0.79^19,24^ and was recommended as a good tool for categorizing patients with varying chances of hospital survival^25^. Consistent with the findings of these studies, in the present study, we report CASPRI score with an AUC of 0.811 for predicting favorable neurological outcomes in cardiac arrest patients who are treated with TTM.

Though the original CASPRI score was developed exclusively for IHCA patients, individual components of the CASPRI score have been associated with the outcomes of TTM, including age^14–16,26^, initial arrest rhythm^14,16,26^, pre-arrest CPC score^14^, arrest location^15,16^, duration of resuscitation^26^, and comorbidities.^14,15^, however, to the best of our knowledge, this is the first study that evaluated the predictive validity of the CASPRI score for patients receiving TTM with concomitant application of ANN algorithms to boost its predictive performance. Our results demonstrate that CASPRI score is also of clinical relevance for patients who received TTM after cardiac arrest. We are cognizant of several other documented models for predicting neurological outcomes for patients receiving or who have received TTM, however, most of them exhibit inferior discrimination power or predictive potential. One such model, the Acute Physiology and Chronic Health Evaluation (APACHE) II score for predicting favorable neurological outcome for patients with OHCA who received TTM exhibited an acceptable discrimination power with an AUC of 0.697^27^. Another model called the Mild Therapeutic Hypothermia score for predicting in-hospital mortality among OHCA patients treated with TTM, reported an AUC of 0.74^28^, while the risk score proposed by Martinell et al. for patients with OHCA receiving TTM yielded AUCs of 0.818–0.842^16^. These scoring systems only demonstrated moderate accuracy, thus, limiting their clinical applications for precise outcome prediction or patient stratification.

More so, understanding the multifactorial nature and complex interplay between baseline conditions, characteristics at the time of cardiac arrest, and the outcomes after TTM, coupled with the challenges associated with obtaining accurate predictions using conventional scoring systems, we exploited the benefits of ANN, a supervised learning algorithm which through emulation of the biological neural architecture, aids identification of relevant predictive markers in the diagnostic task, determines nonlinear data relationships, enhances data interpretation, and informs the design of more efficient diagnostic and predictive models^29–32^. Against this background, our generated ANN-boosted predictive model exhibited high AUC with good accuracy, precision, sensitivity, and specificity, highlighting the applicability of machine learning algorithms to improve the performance and accuracy of CASPRI-based predictive models. Our finding is particularly interesting and clinically relevant because literature review reveals that only few studies have explored the use of machine-learning algorithms to predict the prognoses of patients treated with TTM. Correspondingly, AUCs of 0.82–0.95 were reported by Andersson et al. who included several clinical variables, clinically accessible, and research-grade biomarkers, as predictors of clinical outcomes for patients with OHCA^33^. Johnsson et al. using a cohort of 932 OHCA patients from 36 medical centers, who were treated with TTM, reported an AUC of 0.891 based on 54 clinical variables, and an AUC of 0.852 when three variables, namely, age, time to ROSC, and first monitored rhythm, were used^15^. More so, a previous study by our team using five clinical predictors in the ANN model demonstrated a good predictive performance and notable discrimination power with an AUC of 0.906 for IHCA patients who received TTM^34^. Our current study demonstrates the ANN-boosted technique can accurately predict the neurological outcomes for cardiac arrest patients who received TTM with an AUC of 0.921.

Compared to earlier mentioned studies that focused exclusively on IHCA or OHCA patients, or used complex clinical and serum biomarkers^15,33,34^, our current findings, taking advantage of the simplicity of the widely known CASPRI score, and using readily accessible patient information, highlight the all-inclusive capability of our ANN-boosted model to stratify patients into prognostic groups (favorable outcome vs unfavorable outcome), regardless of cardiac arrest location (IHCA and OHCA). The high AUC value of current study connotes enhanced capability and feasibility of the ANN-boosted CASPRI model with generic predictors to predict the outcome in cardiac arrest patients treated with TTM. The accuracy of clinical predictions can be critical in assisting clinical decision-making for rapid implementation of post-resuscitation therapies. Based on the sensitivity analysis of our ANN model, we also ranked the predictive variables according to their prognostic relevance in patients with cardiac attack who were treated with TTM. Thus, we proffer an ANN-based predictive model with improved predictive performance, that is relatively superior to other conventional statistical approaches or preexisting predictive scoring systems. This ANN-based model is clinically feasible and might further provide the information on the selection of patients who would potentially benefit from TTM treatments.

As with studies of this nature, the present study has some limitations. First, this is a retrospective observational study comprising a relatively small sample of patients who received TTM after successful resuscitation from a cardiac arrest. The limited sample size may restrict the generalizability of current model to a broader population with variable characteristics and prevent complete exclusion of the possibility of model overfitting. Therefore, a large multicenter multi-ethnic cohort with a wide range of clinical and molecular characteristics is required to represent the disease population and validate our results. Second, there was a lack of randomization into TTM or non-TTM groups in current study. The restriction of enrollment to those who received TTM limits the application and generalizability of the current model. Third, the dataset used in the current study did not include the information on interventions in place at the time of cardiac arrest, such as mechanical ventilation, thus, the generated models consisting of 10 predictor variables does not completely represent the CASPRI score that comprised 11 variables. Fourth, previous studies have demonstrated that the time to cooling initiation, time to target temperature, and different cooling methods are associated with neurological outcomes^34–38^. Our proposed CASPRI score-derived ANN-based model, incorporating patients’ clinical characteristics, did not contain data from the resuscitation attempt period, or about the different cooling methods. While this may be considered a limitation to the generalizability of the current neurological outcome predictive model, such consideration must be rightly contextualized in the conclusion of Aitor Uribarri et al.^35^, that “although the speed of cooling initiation and the time to reach target temperature may play a role, its influence on prognosis seems to be less important”. Lastly, there is currently no published data on the validity and reliability of retrospectively determined pre-arrest CPC scores. Further study is required to evaluate pre-arrest CPC measurement characteristics and help interpret the potential limitations or biases of assessments of neurologic status before cardiac arrest.

## Conclusions

Our study further validates the CASPRI score as a prognosticator of functional neurological outcomes for patients who receive TTM after cardiac arrest. The predictive accuracy was significantly improved after applying ANN algorithm. The generated ANN-boosted, CASPRI-based model exhibits good outcome prediction performance. Results documented herein are potentially applicable in clinical settings to facilitate outcome prediction and decision-making to formulate individualized post-resuscitation therapeutic plans.

## Supplementary Information

Supplementary Table S1.

Supplementary Table S2.

## Data Availability

The datasets used and/or analyzed during the current study are available from the corresponding author on reasonable request.

## References

[1] Benjamin EJ (2019). Heart disease and stroke statistics-2019 update: A report from the American Heart Association. Circulation.

[2] Schluep M, Gravesteijn BY, Stolker RJ, Endeman H, Hoeks SE (2018). One-year survival after in-hospital cardiac arrest: A systematic review and meta-analysis. Resuscitation.

[3] Myat A, Song KJ, Rea T (2018). Out-of-hospital cardiac arrest: Current concepts. Lancet (London, England).

[4] Girotra S, Chan PS, Bradley SM (2015). Post-resuscitation care following out-of-hospital and in-hospital cardiac arrest. Heart (British Cardiac Society).

[5] Marcus EL, Chigrinskiy P, Deutsch L, Einav S (2021). Age, pre-arrest neurological condition, and functional status as outcome predictors in out-of-hospital cardiac arrest: Secondary analysis of the Jerusalem Cohort Study data. Arch Gerontol. Geriatr..

[6] Nichol G, Guffey D, Stiell IG, Leroux B, Cheskes S, Idris A (2015). Post-discharge outcomes after resuscitation from out-of-hospital cardiac arrest: A ROC PRIMED substudy. Resuscitation.

[7] Laver S, Farrow C, Turner D, Nolan J (2004). Mode of death after admission to an intensive care unit following cardiac arrest. Intensive Care Med..

[8] O’Leary MJ (2005). Comment on "Mode of death after admission to an intensive care unit following cardiac arrest" by Laver et al. Intensive Care Med..

[9] Mild therapeutic hypothermia to improve the neurologic outcome after cardiac arrest. *N. Engl. J. Med.* **346**, 549–556. 10.1056/NEJMoa012689 (2002). 10.1056/NEJMoa012689 11856793

[10] Bernard SA (2002). Treatment of comatose survivors of out-of-hospital cardiac arrest with induced hypothermia. N. Engl. J. Med..

[11] Song SS, Lyden PD (2012). Overview of therapeutic hypothermia. Curr. Treat. Options. Neurol..

[12] Chiu WT (2020). Post-cardiac arrest care and targeted temperature management: A consensus of scientific statement from the Taiwan Society of Emergency & Critical Care Medicine, Taiwan Society of Critical Care Medicine and Taiwan Society of Emergency Medicine. J. Formosan Med. Assoc..

[13] Hawkes MA, Rabinstein AA (2019). Neurological prognostication after cardiac arrest in the era of target temperature management. Curr. Neurol. Neurosci. Rep..

[14] Chang HC (2021). Factors affecting outcomes in patients with cardiac arrest who receive target temperature management: The multi-center TIMECARD registry. J. Formosan Med. Assoc..

[15] Johnsson J (2020). Artificial neural networks improve early outcome prediction and risk classification in out-of-hospital cardiac arrest patients admitted to intensive care. Crit. Care (Lond. Engl.).

[16] Martinell L (2017). Early predictors of poor outcome after out-of-hospital cardiac arrest. Crit. Care (Lond. Engl.).

[17] Golan E (2014). Predicting neurologic outcome after targeted temperature management for cardiac arrest: Systematic review and meta-analysis. Crit. Care Med..

[18] Chan PS (2012). A validated prediction tool for initial survivors of in-hospital cardiac arrest. Arch. Intern. Med..

[19] Wang CH (2018). Validation of the Cardiac Arrest Survival Post-resuscitation In-hospital (CASPRI) score in an East Asian population. PLoS ONE.

[20] Perkins GD (2015). Cardiac arrest and cardiopulmonary resuscitation outcome reports: update of the Utstein Resuscitation Registry Templates for Out-of-Hospital Cardiac Arrest: A statement for healthcare professionals from a task force of the International Liaison Committee on Resuscitation (American Heart Association, European Resuscitation Council, Australian and New Zealand Council on Resuscitation, Heart and Stroke Foundation of Canada, InterAmerican Heart Foundation, Resuscitation Council of Southern Africa, Resuscitation Council of Asia); and the American Heart Association Emergency Cardiovascular Care Committee and the Council on Cardiopulmonary, Critical Care, Perioperative and Resuscitation. Circulation.

[21] Grossestreuer AV (2016). Inter-rater reliability of post-arrest cerebral performance category (CPC) scores. Resuscitation.

[22] Rittenberger JC, Raina K, Holm MB, Kim YJ, Callaway CW (2011). Association between Cerebral Performance Category, Modified Rankin Scale, and discharge disposition after cardiac arrest. Resuscitation.

[23] Chawla NV, Bowyer KW, Hall LO, Kegelmeyer WP (2002). SMOTE: Synthetic minority over-sampling technique. J. Artif. Int. Res..

[24] Tsai JC, Ma JW, Liu SC, Lin TC, Hu SY (2021). Cardiac Arrest Survival Post-resuscitation In-hospital (CASPRI) score predicts neurological favorable survival in emergency department cardiac arrest. J. Clin. Med..

[25] Andersen LW, Holmberg MJ, Berg KM, Donnino MW, Granfeldt A (2019). In-hospital cardiac arrest: A review. JAMA.

[26] Su PI (2020). Improvement of consciousness before initiating targeted temperature management. Resuscitation.

[27] Kim SI (2018). APACHE II score immediately after cardiac arrest as a predictor of good neurological outcome in out-of-hospital cardiac arrest patients receiving targeted temperature management. Acute Crit. Care.

[28] Kołtowski Ł (2021). Predicting survival in out-of-hospital cardiac arrest patients undergoing targeted temperature management: The Polish Hypothermia Registry Risk Score. Cardiol. J..

[29] Jiang F (2017). Artificial intelligence in healthcare: Past, present and future. Stroke Vasc. Neurol..

[30] Amato F (2013). Artificial neural networks in medical diagnosis. J. Appl. Biomed..

[31] Chung C-C, Chan L, Bamodu OA, Hong C-T, Chiu H-W (2020). Artificial neural network based prediction of postthrombolysis intracerebral hemorrhage and death. Sci. Rep..

[32] Chung CC, Bamodu OA, Hong CT, Chan L, Chiu HW (2021). Application of machine learning-based models to boost the predictive power of the SPAN index. Int. J. Neurosci..

[33] Andersson P (2021). Predicting neurological outcome after out-of-hospital cardiac arrest with cumulative information; development and internal validation of an artificial neural network algorithm. Crit. Care.

[34] Chung CC (2021). Identifying prognostic factors and developing accurate outcome predictions for in-hospital cardiac arrest by using artificial neural networks. J. Neurol. Sci..

[35] Uribarri A (2015). Impact of time to cooling initiation and time to target temperature in patients treated with hypothermia after cardiac arrest. Eur. Heart J. Acute Cardiovasc. Care.

[36] Lee BK (2017). Relationship between timing of cooling and outcomes in adult comatose cardiac arrest patients treated with targeted temperature management. Resuscitation.

[37] Calabró L (2019). Effect of different methods of cooling for targeted temperature management on outcome after cardiac arrest: a systematic review and meta-analysis. Crit. Care.

[38] Bartlett ES (2020). Systematic review and meta-analysis of intravascular temperature management vs surface cooling in comatose patients resuscitated from cardiac arrest. Resuscitation.

