# Supplementary material for: Artificial neural network-boosted Cardiac Arrest Survival Post-Resuscitation In-hospital (CASPRI) score accurately predicts outcome in cardiac arrest patients treated with targeted temperature management
Source: Sci Rep. 2022 May 4;12:7254. doi: 10.1038/s41598-022-11201-z (PMC9068683; doi:10.1038/s41598-022-11201-z)
Supplement: Supplementary file 1 — Supplementary Table S1. [file 41598_2022_11201_MOESM1_ESM.pdf]

Table S1. Cardiac Arrest Survival Postresuscitation In-hospital (CASPRI) Score

Predictor	Points
1. Age group, y	
<50	0
50-59	0
60-69	1
70-79	2
≧80	4
2. Initial arrest rhythm	
VF/VT time to defibrillation	
≦2 minutes	0
3 minutes	0
4-5 minutes	2
>5 minutes	3
Pulseless electrical activity	6
Asystole	7
3. Prearrest CPC score	
1	0
2	2
3	9
≧4	9
4. Hospital location	
Telemetry unit	0
Intensive care	1
Nonmonitored unit	3
5. Duration of resuscitation, minutes	
<2	0
2-4	0
5-9	3
10-14	5
15-19	6
20-24	6
25-29	6
≧30	8
Factors present prior to arrest	
6. Mechanical ventilation	3
7. Renal insufficiency	2
8. Hepatic insufficiency	4
9. Sepsis	3
10. Malignant disease	4
11. Hypotension	3

VF, ventricular fibrillation; VT, ventricular tachycardia; CPC, cerebral performance score
