# Supplementary material for: Artificial neural network-boosted Cardiac Arrest Survival Post-Resuscitation In-hospital (CASPRI) score accurately predicts outcome in cardiac arrest patients treated with targeted temperature management
Source: Sci Rep. 2022 May 4;12:7254. doi: 10.1038/s41598-022-11201-z (PMC9068683; doi:10.1038/s41598-022-11201-z)
Supplement: Supplementary file 2 — Supplementary Table S2. [file 41598_2022_11201_MOESM2_ESM.pdf]

Table S2. Comparison between the variables before and after SMOTE oversampling

Variables of CASPRI score	Favorable outcome (n = 117)	SMOTE generated cases (n = 336)	*p*-value	OR (95% CI)
Age (years)	58.1 ± 16.6	58.0 ± 15.2	0.932	1.00 (0.99–1.01)†
Initial cardiac arrest rhythm, n (%)			0.950	
VF/Pulseless VT	79 (67.5)	239 (71.1)		
Pulseless electrical activity	30 (26.6)	81 (24.1)		
Asystole	8 (6.8)	16 (4.8)		
Pre-arrest CPC score	1.04 ± 0.20	1.02 ± 0.13	0.340	0.47(0.14–1.66)†
Arrest location, n (%)				
OHCA	97 (82.9)	284 (84.5)	0.662	0.89 (0.50–1.56)
IHCA	20 (17.1)	52 (15.5)	0.743	
Telemetry unit	14 (12.0)	40 (11.9)		
Intensive care unit	1 (0.9)	3 (0.9)		
Non-monitored unit	5 (4.3)	9 (2.7)		
Duration of resuscitation (min)	21.5 ± 21.0	20.6 ± 16.4	0.685	1.00 (0.98–1.01)†
MAP at ROSC (mmHg)	104.3 ±29.8	102.6 ± 26.4	0.585	1.00(0.99–1.01)†
Comorbidities, n (%)				
Renal insufficiency	18 (15.4)	45 (13.4)	0.642	1.18 (0.65–2.13)
Hepatic insufficiency	1 (0.9)	1 (0.3)	0.450	2.89 (0.18–46.5)
Sepsis	3 (2.6)	5 (1.5)	0.431	1.74 (0.41–7.41)
Malignancy	7 (6.0)	16 (4.8)	0.627	1.27 (0.51–3.18)

CASPRI, Cardiac Arrest Survival Postresuscitation In-hospital; CI, confidence interval; CPC, cerebral performance category; IHCA, in-hospital cardiac arrest; MAP, mean arterial pressure; OHCA, out-of-hospital cardiac arrest; OR, odds ratio; ROSC, return of spontaneous circulation; SMOTE, Synthetic Minority Over-sampling Technique;VF, ventricular fibrillation; VT, ventricular tachycardia. †Odds ratio of per unit changes.
